# Association Between Distance to an Abortion Facility and Abortion or Pregnancy Outcome Among a Prospective Cohort of People Seeking Abortion Online

**DOI:** 10.1001/jamanetworkopen.2022.12065

**Published:** 2022-05-13

**Authors:** Elizabeth A. Pleasants, Alice F. Cartwright, Ushma D. Upadhyay

**Affiliations:** 1 School of Public Health, University of California, Berkeley; 2 Department of Maternal and Child Health, Gillings School of Global Public Health, University of North Carolina at Chapel Hill; 3 Carolina Population Center, University of North Carolina at Chapel Hill; 4 Advancing New Standards in Reproductive Health, Department of Obstetrics, Gynecology and Reproductive Sciences, University of California, San Francisco, Oakland

## Abstract

**Importance:**

Many people face barriers to abortion care, including long distances to an abortion facility.

**Objectives:**

To investigate the association of distance to the nearest abortion facility with abortion or pregnancy outcome.

**Design, Setting, and Participants:**

This cohort study was conducted using data from the Google Ads Abortion Access study, a prospective cohort study of individuals considering abortion recruited between August 2017 and May 2018. Individuals from 50 states and Washington, District of Columbia, who were pregnant and considering abortion based on self-report were recruited online using a stratified sampling technique. Participants completed online baseline and 4-week follow-up surveys. Data were analyzed between May and August 2021.

**Exposures:**

Driving distance to an abortion facility calculated from participant zip code and grouped into 4 categories (<5 miles, 5-24 miles, 25-49 miles, and ≥50 miles).

**Main Outcomes and Measures:**

Abortion or pregnancy outcome reported at 4-week follow-up, categorized as had an abortion, still seeking an abortion, or planning to continue pregnancy. Other measures included reported experience of 8 distance-related barriers to abortion, such as having to gather money for travel expenses and having to keep the abortion a secret.

**Results:**

Among 1485 pregnant individuals considering abortion who completed the baseline survey and provided contact information, 1005 individuals completed follow-up (follow-up rate, 67.7%) and 856 participants were included in the analytic sample (443 individuals ages 25-34 years [51.8%]; 208 Black individuals [24.3%]; 101 Hispanic or Latinx individuals [11.8%], and 468 White individuals [54.8%]). Most participants had at least some college education (474 individuals [55.5%]). Distance to an abortion facility was less than 5 miles for 233 individuals (27.2%), 5 to 24 miles for 373 individuals (43.6%), 25 to 49 miles for 85 individuals (9.9%), and 50 or more miles for 165 individuals (19.3%) (mean [SD] distance = 28.3 [43.8] miles). Most participants reported at least 1 distance-related barrier (763 individuals [89.1%]), with a mean of 3.3 barriers (95% CI, 3.2-3.5 barriers) reported. For 7 of 8 distance-related barriers, an increased percentage of participants living farther from an abortion facility reported the barrier compared with participants living less than 5 miles from a facility; for example, 61.8% (95% CI, 53.5%-69.4%) of individuals living less than 5 miles reported having to gather money for travel expenses, while 81.2% (95% CI, 70.8%-88.5%; *P* = .002) of those living 25 to 49 miles and 75.8% (95% CI, 69.9%-81.0%; *P* = .02) of those living 50 or more miles from a facility reported this barrier. At follow-up, participants living 50 or more miles from a facility had higher odds of still being pregnant and seeking abortion (adjusted odds ratio [aOR] = 2.07; 95% CI, 1.35-3.17; *P* = .001) or planning to continue pregnancy (aOR = 1.96; 95% CI, 1.06-3.63; *P* = .03) compared with participants living within 5 miles.

**Conclusions and Relevance:**

This study found that greater distance from an abortion facility was associated with delays in obtaining abortion care and inability to receive abortion care. These findings suggest that innovative approaches to abortion provision may be needed to mitigate outcomes associated with long distances to abortion facilities.

## Key Points

**Question:**

Is distance to the nearest abortion facility associated with abortion or pregnancy outcome among individuals considering abortion?

**Findings:**

In this cohort study of 856 individuals considering abortion and seeking abortion care online, living 50 miles or more from an abortion facility was associated with still being pregnant (still seeking an abortion or planning to continue pregnancy) 4 weeks later.

**Meaning:**

This finding suggests that travel distance to abortion facilities may be an important barrier to abortion access in the US.

## Teaser

This cohort study investigates the association between distance to the nearest abortion facility and abortion and pregnancy outcome among individuals seeking abortion care and information online.

## Introduction

Abortion is a common pregnancy outcome in the United States and an essential health service, yet many people must travel long distances to reach an abortion facility. As of 2017, 89% of counties within the US did not have a facility providing abortion.^1^ Myriad state laws across the US make it harder to obtain an abortion. An unprecedented number of increasingly aggressive restrictions have been enacted in recent years, including the 6-week abortion ban in Texas and laws in 26 states that will certainly or likely ban abortion if the US Supreme Court overturns *Roe v Wade*.^2,3^ State-level abortion restrictions also influence the geographic distribution of facilities, given that these restrictions may be associated with facilities closing and dissuade new facilities from opening, often where needed most.^4,5,6,7,8^

Most people seeking abortion have to travel less than 25 miles one way to reach a provider, but many individuals have to travel 50 or more miles.^9,10^ Between 2011 and 2017, the number of abortion-providing facilities in the US decreased by almost 8%, with the largest decreases seen in the Midwest and South.^1,11^ Abortion facility closures reduce access to abortion by significantly increasing required travel distances for individuals to reach an abortion-providing clinician.^4,12,13^ Spatial disparities in abortion access have been found across the US, with women who are White, living in the South and Midwest, living in rural areas, and seeking later abortions having to travel significantly farther to reach a clinician.^9,10,14,15,16,17^

Traveling long distances for abortion care has been associated with burdens, including incurring transportation costs, needing time off work, needing to disclose an abortion, and needing to find child care.^4,18,19,20,21^ Abortion facility closures are often followed by significantly lower abortion rates overall and increased travel distances to facilities, out-of-pocket costs, birth rates, mean pregnancy duration at the time of abortion, and delays in accessing care.^4,6,7,8,22,23,24^ Delays in care contribute to an ongoing cycle of increased pregnancy durations and more expensive abortions, necessitating more fundraising.^25^ National studies^25,26,27^ from 2014 to 2021 using county-level data and projected changes in abortion rates if *Roe v Wade* is overturned found that decreased supply of abortion services and increased travel distances to facilities were associated with lower abortion rates and delayed access to abortion. These studies, however, did not include individual-level data.

An analysis using data from the Google Ads Abortion Access Study^28^ found that almost half of people considering abortion had obtained one by 4 weeks of follow-up (48%), while 32% were still seeking an abortion and 20% had decided to continue their pregnancy.^29^ Given these findings and the challenges posed to abortion access by increased travel distance, we sought to explore the association of distance to an abortion-providing facility with abortion attainment in this sample. This study had the following objectives: (1) to describe individual factors associated with greater travel distances to abortion facilities and the related barriers to obtaining abortion care reported and (2) to explore associations between distance to an abortion facility and abortion or pregnancy outcome.

## Methods

The University of California, San Francisco, Institutional Review Board approved this cohort study. Participants provided electronic written consent. This study followed Strengthening the Reporting of Observational Studies in Epidemiology (STROBE) reporting guideline.

### Study Design

Data were from the Google Ads Abortion Access study,^28^ a prospective cohort study conducted to investigate barriers to abortion care among currently pregnant individuals in the US seeking abortion information and services online. Data were collected between August 2017 and May 2018; detailed methods for this study are published elsewhere.^28^ In short, individuals searching via Google for specific abortion-related queries (eg, “abortion clinic near me”) were recruited through advertisements to obtain a state-stratified sample for all 50 states and Washington, District of Columbia. Individuals who clicked the advertisement were screened for eligibility. To qualify, they needed to be currently pregnant and considering abortion; eligibility was not restricted based on age. Eligible individuals completed a baseline survey and provided contact information (email and/or phone number) for follow-up. The study team monitored recruitment by state to ensure that responses were not disproportionately obtained from states with larger populations. A range of 5 to 20 participants was enrolled from each location.^28^

Participants were contacted 4 weeks after the baseline survey via email or text message with an invitation to complete the follow-up survey. Those who had a miscarriage, live birth, or other pregnancy outcome (eg, were never pregnant) after baseline were not eligible for follow-up. All eligible participants who completed the follow-up survey were sent a $50 electronic gift card. Data were analyzed between May and August 2021.

### Data

The primary outcome of interest was self-reported abortion or pregnancy at 4-week follow-up, categorized as had an abortion, pregnant and still seeking an abortion, or pregnant and planning to continue the pregnancy (see survey questions in the eAppendix in the Supplement). The primary exposure of interest was driving distance for the participant to reach an abortion facility. This measure was calculated in ArcGIS online (Esri)^30^ using the driving distance analysis function.^31^ The centroid of each participant’s self-reported zip code at baseline was used to approximate residence based on 2010 US Census ZIP Code Tabulation Areas.^32^ Addresses for abortion facilities were obtained from the Advancing New Standards in Reproductive Health Abortion Facility Database, which contained data on all publicly advertised US abortion facilities generated through a systematic online search in 2017.^33,34^ Locations were compiled for each facility and participant by latitude and longitude, and ArcGIS was used to create a location and density map of facilities and participants. Distances between participants and nearest abortion facility were calculated and categorized into 4 categories for this analysis: less than 5 miles, 5 to 24 miles, 25 to 49 miles, and 50 miles or more.

Key covariates collected at baseline included various sociodemographic characteristics, abortion history, residence, and pregnancy duration. Socioeconomic status was assessed using a proxy measure: difficulty meeting basic needs in the prior 12 months. Self-reported race and ethnicity served as a proxy for the sociocultural experiences of individuals, including different barriers to abortion access that individuals may face based on the historic privilege or disadvantage experienced by their racial or ethnic group. Response options for ethnicity were yes or no for Hispanic or Latina (hereafter, *Latinx*), and response options for racial and ethnic groups were American Indian or Alaska Native, Asian, Black or African American, Native Hawaiian or other Pacific Islander, White, other (consisting of an open-ended text box in which participants could specify their race or ethnicity), and 2 or more races/multiracial (consisting of a checkbox with an optional open-ended text box to specify races and ethnicities). Individuals who responded yes to Hispanic or Latina and were categorized as "Hispanic or Latinx" regardless of additional races they selected. Participants from racial groups with small sample sizes in the analytic sample (ie, <5%) were combined into a single group for this analysis. Highest education level completed was determined based on report of highest grade of school completed by a participant, categorized as high school graduate or less or some college or more. Health insurance type was determined based on participant report of having private insurance, public insurance (Medicaid, Medicare, or state health exchange coverage), no health insurance coverage, or not being sure if they had health insurance. The state policy environment for each participant was determined based on access ratings by NARAL Pro-Choice America to reflect restrictiveness of state abortion laws based on pro- and anti-choice legislation in effect in 2018, including policies related to contraception and abortion access, categorized as protected access, some access, and restricted access.^29,35^ Pregnancy duration was calculated from participant responses to a series of questions ordered from most to least precise. First, we asked the date of last menstrual period; if unknown, we asked for months since last menstruation, and if that was unknown, we asked for estimated months pregnant.

At follow-up, participants were asked questions about their experiences trying to access an abortion. They were asked to rate how easy or hard it is for someone to get an abortion in the area where they live on a 5-point Likert scale (from 1, very easy, to 5, very hard). Participants were also asked a series of questions about factors that made it difficult for them to get an abortion (eg, “I had to arrange for transport to the clinic”) and asked to select all factors that applied. Free text responses to an *other* barrier response option were reviewed by 2 research assistants and categorized within the listed barriers as appropriate. For this analysis, only barriers related to the ability to physically reach an abortion facility were included, categorized as a set of distance-related barriers. The distance-related barriers included were (1) “I didn’t know where to get an abortion,” (2) “The distance I had to travel to the clinic made it hard,” (3) “I had to arrange for childcare or care for other family member,” (4) “I had to make multiple trips to the clinic,” (5) “I had to arrange for transport to the clinic,” (6) “I had to get time off work/school,” (7) “I had to keep the abortion a secret,” and (8) “I had to gather money for travel expenses.”

At follow-up, participants who had obtained an abortion were asked if they could easily find information online about where to get an abortion. They were also asked if they found the facility where they got their abortion through their online search.

### Statistical Analysis

We first described baseline characteristics of participants by their categorized distance to an abortion facility and compared them using χ^2^ tests or *t* tests. We analyzed reported barriers to abortion access, summarizing participant scale ratings for ease of access to abortion. Next, we summarized responses on ease of finding abortion information online and if participants found the clinic where they got their abortion online among participants who reported having an abortion at follow-up. We then used separate binary mixed-effects logistic regression models to explore associations between distance to an abortion facility and 8 individual barriers to abortion access while controlling for state clustering. Based on these models, we estimated the proportions of each distance-related barrier and examined the association between reported barriers and distance to an abortion facility by group (ie, <5 miles, 5-24 miles, 25-49 miles, and ≥50 miles).

Finally, we used a multinomial logistic regression model to examine the association between distance to the nearest abortion facility and the 3-category abortion or pregnancy outcome, adjusting for the following baseline sociodemographic characteristics hypothesized a priori to be associated with the dependent variable: age, race and ethnicity, highest education level completed, insurance type, difficulty meeting basic needs, previous abortion, and duration of pregnancy. Standard errors in all models accounted for clustering at the state level as a random effect. Results of all models are expressed with adjusted odds ratios (aORs) and 95% CIs. All statistical tests were 2-tailed, with significance set at P < .05. Analyses were done using Stata/IC statistical software version 15.1 (StataCorp).

## Results

Participants who had complete data on abortion or pregnancy outcome and abortion access barriers at follow-up constituted the final analytic sample. As described previously,^28^ 1485 people completed the baseline survey and provided contact information, among whom 3 individuals were from outside the US and 21 individuals completed the survey more than once and were therefore excluded from analysis. Among 1005 participants who completed the follow-up survey (follow-up rate, 67.7%), 131 individuals reported an ineligible or unknown pregnancy outcome (ie, stillbirth, miscarriage, live birth, or unknown outcomes) and 18 individuals did not respond to all questions on distance-related barriers to abortion access, leaving a final analytic sample of 856 participants.

Descriptive information for participants is presented in Table 1. Most eligible participants were ages 25 to 34 years (443 individuals [51.8%]). There were 78 individuals who were Asian or multiracial or had other race or ethnicity (9.1%); 208 Black individuals (24.3%); 101 Hispanic or Latinx individuals (11.8%), and 468 White individuals (54.8%). Most participants had at least some college education (474 individuals [55.5%]) and public health insurance (ie, Medicaid, Medicare, or state exchange; 446 individuals [52.1%]), and almost half of participants had difficulty meeting basic needs in the year prior to baseline (395 individuals [46.1%]). A portion of participants resided in each region of the US, with the largest proportion in the South (274 individuals [32.0%]) and the smallest in the Northeast (158 individuals [18.5%]), and most participants lived in states with restricted access to abortion (515 individuals [60.2%]).

**Table 1.** Baseline and Follow-up Characteristics Among Participants With Complete Follow-up

Characteristic	Participants, No. (%)					*P* value
	Total (N = 856)	<5 Miles to facility (n = 233 [27.2%])	5-24 Miles to facility (n = 373 [43.6%])	25-49 Miles to facility (n = 85 [9.9%])	≥50 Miles to facility (n = 165 [19.3%])^a^	
**Baseline characteristic**						
Age, y						
<25	294 (34.3)	75 (32.2)	112 (32.7)	34 (40.0)	63 (38.2)	.28
25-34	443 (51.8)	127 (54.5)	194 (52.0)	36 (42.4)	86 (52.1)	
≥35	119 (13.9)	31 (13.3)	57 (15.3)	15 (17.6)	16 (9.7)	
Race and ethnicity						
Asian, multiracial, or other^b^	78 (9.1)	27 (11.6)	31 (8.3)	6 (7.1)	14 (8.5)	.002
Black or African American	208 (24.3)	73 (31.3)	89 (23.9)	13 (15.3)	33 (20.0)	
Hispanic or Latinx	101 (11.8)	36 (15.5)	40 (10.7)	9 (10.6)	16 (9.7)	
White	469 (54.8)	97 (41.6)	213 (57.1)	57 (67.1)	102 (61.8)	
Education						
≤High school graduate	382 (44.6)	100 (42.9)	160 (42.9)	39 (45.9)	83 (50.3)	.40
Some college, college, or professional degree	474 (55.5)	133 (57.1)	213 (57.1)	46 (54.1)	82 (49.7)	
Health insurance						
None or not sure	197 (23.0)	40 (17.2)	91 (24.4)	23 (27.1)	43 (26.1)	.048
Private	213 (24.9)	61 (26.2)	104 (27.9)	18 (21.1)	30 (18.2)	
Medicaid, Medicare, or state exchange	446 (52.1)	132 (56.7)	178 (47.7)	44 (51.8)	92 (55.8)	
Had difficulty meeting basic needs in the last year	395 (46.1)	96 (41.2)	177 (47.5)	43 (50.6)	79 (47.9)	.33
Had previous abortion	254 (29.7)	85 (36.5)	110 (29.5)	18 (21.2)	41 (24.8)	.02
Baseline pregnancy duration, wk						
≤10.0	678 (79.2)	182 (78.1)	300 (80.4)	63 (74.1)	133 (80.6)	.40
10.1-14.0	101 (11.8)	34 (14.6)	42 (11.3)	10 (11.8)	15 (9.1)	
≥14.1	60 (7.0)	13 (5.6)	24 (6.4)	8 (9.4)	15 (9.1)	
Missing	17 (2.0)	4 (1.7)	7 (1.9)	4 (4.7)	2 (1.2)	
US census–defined region of residence						
Northeast	158 (18.5)	63 (27.0)	78 (20.9)	8 (9.4)	9 (5.5)	<.001
South	274 (32.0)	53 (22.7)	110 (29.5)	36 (42.4)	75 (45.5)	
Midwest	222 (25.9)	57 (24.5)	84 (22.5)	28 (32.9)	53 (32.1)	
West	202 (23.6)	60 (25.8)	101 (27.1)	13 (15.3)	28 (17.0)	
Restrictiveness of state by NARAL rating						
Protected access	233 (27.2)	92 (39.5)	109 (29.2)	15 (17.6)	17 (10.3)	<.001
Some access	108 (12.6)	33 (14.2)	59 (15.8)	6 (7.1)	10 (6.1)	
Restrictive access	515 (60.2)	108 (46.4)	205 (55.0)	64 (75.3)	138 (83.6)	
**Follow-up characteristic**						
Pregnancy or abortion outcome group						
Had abortion	409 (47.8)	120 (51.5)	187 (50.1)	44 (1.82)	58 (35.2)	.04
Pregnant and seeking abortion	279 (32.6)	69 (29.6)	115 (30.8)	27 (31.8)	68 (41.2)	
Continuing pregnancy	168 (19.6)	44 (18.9)	71 (19.0)	14 (16.5)	39 (23.6)	
≥1 Distance-related barrier reported	763 (89.1)	203 (87.1)	324 (86.9)	82 (96.5)	154 (93.3)	.01
No. of distance-related barriers reported, mean (95% CI)	3.3 (3.2-3.5)	2.8 (2.5-3.0)	3.1 (2.9-3.3)	4.2 (3.8-4.6)	4.2 (3.9-4.5)	<.001
Score on response to ease of getting abortion in area, mean (95% CI)^c^	3.2 (3.1-3.3)	2.7 (2.6-2.9)	3.0 (2.8-3.1)	3.5 (3.2-3.8)	4.0 (3.9-4.2)	<.001

^a^ Includes 2 people in Hawaii who would have to fly the straight-line distance, given that there was no facility on their island.

^b^ Other race and ethnicity includes Native Hawaiian or Pacific Islander and American Indian or Alaska Native, combined with Asian and multiracial for this analysis owing to small sample sizes for all groups (<5% of analytic sample).

^c^ Score is on a scale of 1 to 5 on following question: “Generally, how easy or hard do you think it is for someone to get an abortion in the area where you live?”

**Figure 1.**
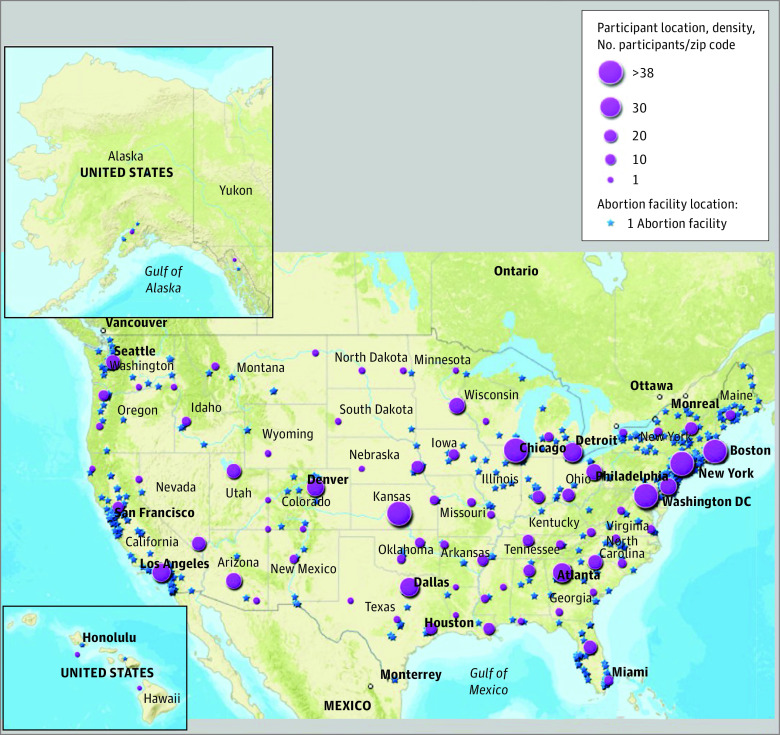
Abortion Facility Locations in 2017 and Density of Participant Locations Map shows locations of 781 abortion facilities and 856 participants, with participant density shown by latitude and longitude.

Abortion facility and participant locations are mapped in Figure 1. Distance to the nearest abortion facility was less than 5 miles for 233 participants (27.2%), 5 to 24 miles for 373 participants (43.6%), 25 to 49 miles for 85 participants (9.9%), and 50 miles or more for 165 participants (19.3%). The median (range) distance to a facility in our sample was 9.6 (<1 to 321.4) miles, and the mean (SD) distance was 28.3 (43.8) miles. Baseline sociodemographic, residence, and pregnancy characteristics and follow-up barriers and outcomes of the study sample are presented in Table 1.

Almost all participants reported at least 1 distance-related barrier to abortion access (763 individuals [89.1%]), with a mean of 3.3 barriers (95% CI, 3.2-3.5 barriers) reported across participants. A greater mean number of barriers were reported by participants living 25 to 49 miles (4.2 barriers [95% CI, 3.8-4.6 barriers]) and 50 miles or more (4.2 barriers [95% CI, 3.9-4.5 barriers]) from a facility compared with those living closer (5-24 miles: 2.8 [95% CI, 2.5-3.0 barriers]; <5 miles: 3.1 barriers [95% CI, , 2.9-3.3 barriers]) (*P* < .001). Participants living farther from an abortion facility more often indicated that it was difficult for people to access an abortion where they live, with a mean scale score of 4.0 (95% CI, 3.9-4.2) among individuals living 50 miles or more from a facility compared with 2.7 (95% CI, 2.6-2.9) among those living less than 5 miles (*P* < .001; scale range: 1-5, very easy to very hard).

**Figure 2.**
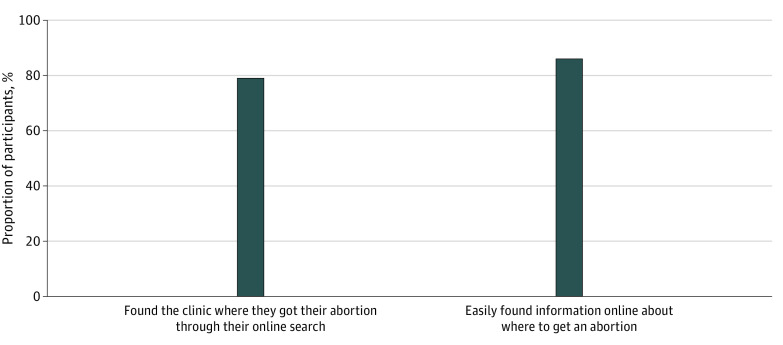
Online Abortion Information-Seeking Behaviors Behaviors are shown among 409 participants who had obtained an abortion by 4-week follow-up.

Among 409 participants who had obtained an abortion by follow-up, most individuals reported that they could easily find information about where to get an abortion online (362 individuals [86.2%]) (Figure 2). Additionally, within the same group, most participants reported having found the location where they got an abortion through an online search (322 individuals [79.0%]). Neither of these was associated with distance to an abortion facility.

**Figure 3.**
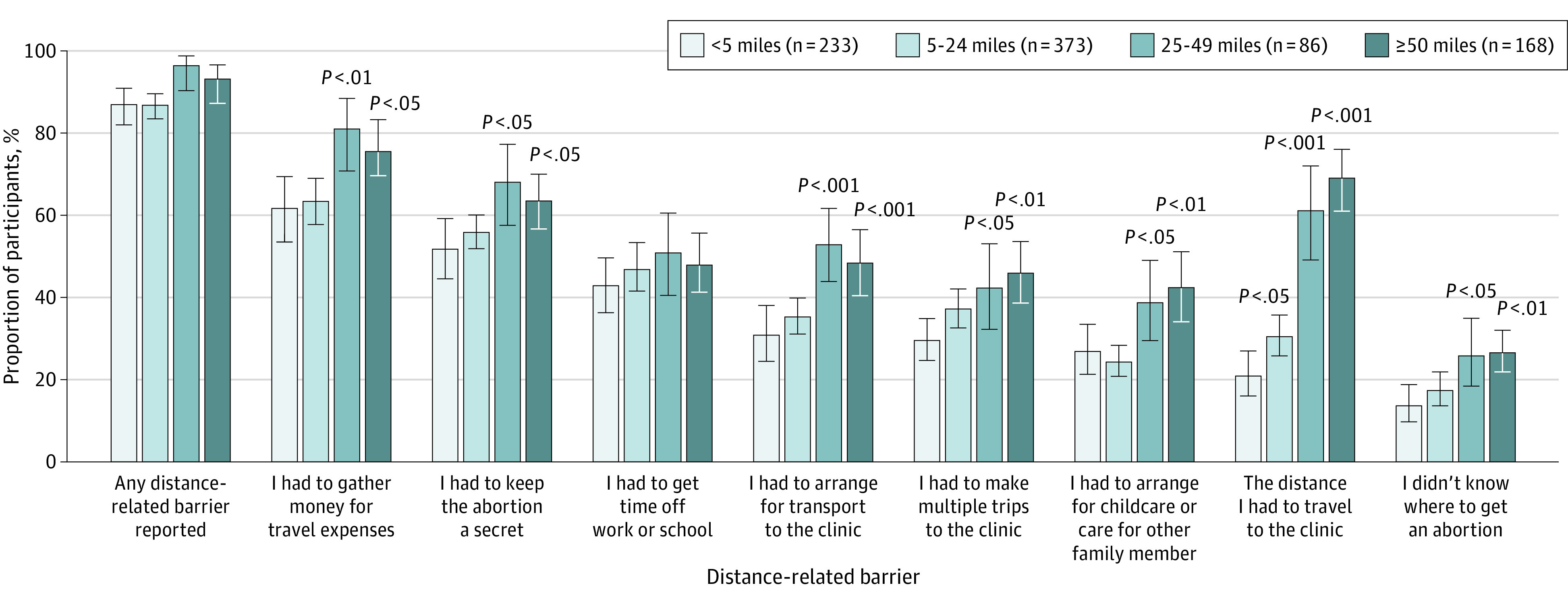
Estimated Proportions of Participants Reporting Distance-Related Barriers to Abortion by Distance Category Estimated proportions are based upon mixed-effects logistic regression models accounting for clustering by respondents’ state of residence. *P* values are for estimated proportions reporting this barrier within each distance group compared with individuals living less than 5 miles from an abortion facility. Whiskers indicate 95% CIs.

Estimated proportions of individual distance-related barriers accounting for state-level clustering are presented in Figure 3. Having to gather money for travel expenses and keep the abortion a secret were the most commonly reported barriers across participant groups by distance to abortion facility. Participants living 25 to 49 miles or 50 miles or more from a facility were significantly more likely to report almost all distance-related barriers than those living less 5 miles from a facility; the exception was having to get time off school or work. For example, 61.8% (95% CI, 53.5%-69.4%) of individuals living less than 5 miles from a facility reported having to gather money for travel expenses, while 81.2% (95% CI, 70.8%-88.5%; *P* = .002) of those living 25 to 49 miles and 75.8% (95% CI, 69.9%-81.0%; *P* = .02) of those living 50 or more miles from a facility reported this barrier. Full results for mixed-effects logistic regression models showing the associations between distance to abortion facility and distance-related barriers are presented in the eTable in the Supplement.

The adjusted model examined the association between distance to facility and abortion or pregnancy outcome. In this model, participants living 50 or more miles from an abortion facility had significantly higher odds of still being pregnant and seeking an abortion (aOR = 2.07; 95% CI, 1.35-3.17; *P* = .001) or planning to continue their pregnancy (aOR = 1.96; 95% CI, 1.06-3.63; *P* = .03) than having had an abortion compared with participants living less than 5 miles from a facility (Table 2).

**Table 2.** Odds of Being Pregnant and Still Seeking Abortion or Continuing Pregnancy at 4 Weeks in Multinomial Logistic Regression^a^

Distance to abortion facility, miles	Pregnant, still seeking abortion, aOR (95% CI)^b^	*P* value	Pregnant, continuing pregnancy, aOR (95% CI)^b^	*P* value
<5	[1 Reference]	NA	[1 Reference]	NA
5-24	1.12 (0.75-1.67)	.58	1.13 (0.70-1.81)	.62
25-49	1.01 (0.55-1.88)	.97	0.85 (0.41-1.76)	.67
≥50^c^	2.07 (1.35-3.17)	.001	1.96 (1.06-3.63)	.03

Abbreviations: aOR, adjusted odds ratio; NA, not applicable.

^a^ Model was adjusted for the following baseline covariates: age, race and ethnicity, education, insurance, difficulty meeting basic needs in the prior year, previous abortion, and duration of pregnancy.

^b^ Reference group was participants who had obtained an abortion by 4-week follow-up.

^c^ Includes 2 people in Hawaii who would have to fly the straight-line distance given that there was no facility on their island.

## Discussion

In this cohort study, we found that participants living far from an abortion facility had 2-fold the odds of still seeking an abortion or planning to continue pregnancy at the 4-week follow-up survey. Previous research found that greater distance to an abortion facility was associated with reduced and delayed access to abortion among individuals recruited at abortion clinics^16,23^ or reduced abortion rates at the county level.^6,7,8,22,26,27^ This study aligns with and expands upon those results, finding that greater travel distance to reach an abortion facility was associated with delays in access and prevention of access to wanted abortion care among a large sample of individuals considering abortion in the US.

Individuals who were White and living in regions with more restrictive abortion laws lived farther from facilities. This is consistent with previous findings on spatial disparities in abortion access by race and region of residence,^9,10,15,16,36^ although we did not find an association between later pregnancy duration at baseline and increased distance to abortion facilities, in contrast to previous research findings.^10,15,36^ This may be associated with this study’s use of web-based recruitment to reach not only patients who had abortions, but also individuals considering abortions. Our study directly explored self-reported access to abortion and found that longer travel distances were associated with delayed access, as evidenced by the increased odds of still seeking a desired abortion at the 4-week follow-up survey among individuals living more than 50 miles from a facility compared with those living within 5 miles of a facility. Living farther from an abortion facility is associated with increased burdens in the process of seeking an abortion, including direct and indirect travel costs, which can pose a particular challenge for individuals with economic disadvantages.^4,18,19,20,21,37^

Innovative approaches to abortion care can help lessen the burdens associated with distance. Medication abortion provision in the US is currently limited by laws in 33 states that require medication abortion administration by a physician^38^ and laws in 19 states that effectively ban telehealth provision.^39,40^ The federal government recently permanently lifted the in-person requirement for the provision of medication abortion, thereby allowing dispensing via telehealth and mail, but individuals in those 19 states cannot avail of this evolution in access.^41,42^ Lifting state restrictions on telehealth could substantially improve access to abortion for people in the US, particularly individuals living 50 miles or more from an abortion facility,^43^ and is supported by current evidence for the safety, acceptability, and feasibility of expanded provision of medication abortion.^44,45,46^ Furthermore, research simulating the association of service delivery innovations with abortion rates found that making telehealth abortion widely available or integrating abortion into primary care would be associated with increased access and reduced unmet need for abortion in the US.^27^

Given increases in the number of state-level abortion restrictions during the past decade and concerns about the continued legality of abortion across the US,^47,48^ travel distances to obtain abortions are likely to increase and be associated with decreases in abortion rates and increases in instances of self-managed abortion, unplanned pregnancy rates, and maternal morbidity and mortality.^19,48^ The outcomes associated with a persistent decline in abortion facilities warrant continued monitoring and research.

### Strengths and Limitations

This analysis has several strengths and limitations. While we were able to determine distance to the nearest abortion facility for participants, proximity to 1 facility does not ensure that this facility meets people’s needs.^9,26,49^ Using the nearest facility to determine travel distance may have underestimated travel distance to obtain desired abortion services. Additionally, this study included a large, multistate sample across all 50 states and Washington, District of Columbia, with a follow-up rate (67.7%) similar to those in facility-based studies.^28^ Despite this, the sample did not provide representative data on distance, distance-related barriers, or abortion and pregnancy outcomes across the US. Rather, our findings provided insights into individual-level factors associated with abortion access among a hard-to-reach population: individuals considering abortion who had not yet reached a facility.

Although this research benefited from efficient internet recruitment of a hard-to-reach population, it was restricted to people seeking abortion information on Google. Recruitment may not have effectively reached all individuals considering abortion, particularly those without internet access or with privacy concerns around seeking abortion information or participating in this study. However, research has found that internet-based recruitment is not necessarily associated with biased measures of association in prospective cohort studies, with similar results found in internet and noninternet sampling approaches.^50^ Differential loss to follow-up may have also contributed to biasing our results, given that there was greater follow-up among participants who were older, White, and more educated and who did not have challenges meeting basic needs. Notably, the only sociodemographic characteristic also associated with longer travel distance was race, with White individuals more often living father from a facility; this outcome, therefore, possibly contributed to an overestimation of travel distance in our sample.

## Conclusions

This analysis found that greater distance to reach a facility was associated with delays to obtaining abortion care and inability to receive desired abortion care. Greater distance was also associated with additional burdens in the abortion-seeking process. These findings suggest that innovative approaches to abortion provision that mitigate outcomes associated with distance to a dedicated abortion facility, including use of telehealth and provision of abortion by a broader range of health care clinicians, should be prioritized for improving abortion access.

## Notes

Supplement. **eAppendix.** Google Ads Abortion Access Study Survey Questions Providing Data Used for Analysis **eTable.** Adjusted Odds Ratios for Association Between Selected Distance-Related Barriers to Abortion Access and Distances to Abortion
