# Supplementary material for: Association Between Distance to an Abortion Facility and Abortion or Pregnancy Outcome Among a Prospective Cohort of People Seeking Abortion Online
Source: JAMA Netw Open. 2022 May 13;5(5):e2212065. doi: 10.1001/jamanetworkopen.2022.12065 (PMC9107030; doi:10.1001/jamanetworkopen.2022.12065)
Supplement: Supplement. — eAppendix. Google Ads Abortion Access Study Survey Questions Providing Data Used for Analysis eTable. Adjusted Odds Ratios for Association Between Selected Distance-Related Barriers to Abortion Access and Distances to Abortion [file jamanetwopen-e2212065-s001.pdf]

Supplemental Online Content

Pleasants EA, Cartwright AF, Upadhyay UD. Association between distance to an abortion facility and abortion or pregnancy outcome among a prospective cohort of people seeking abortion online. *JAMA Netw Open.* 2022;5(5):e2212065. doi:10.1001/jamanetworkopen.2022.12065

eAppendix. Google Ads Abortion Access Study Survey Questions Providing Data Used for Analysis

eTable. Adjusted Odds Ratios for Association Between Selected Distance-Related Barriers to Abortion Access and Distances to Abortion

This supplemental material has been provided by the authors to give readers additional information about their work.

© 2022 Pleasants EA et al. *JAMA Network Open.*

eAppendix. Google Ads Abortion Access Study Survey Questions Providing Data Used for Analysis

1. Are you pregnant right now?
   - ☐ Yes
   - ☐ No
   - ☐ Not sure

2. What happened with your most recent pregnancy? (As a reminder, your responses are completely confidential.)
   - ☐ I had an abortion procedure done by a clinician in a clinic or doctor's office
   - ☐ I took abortion pills that I got from a clinic, doctor or nurse
   - ☐ I took abortion pills that I got from the internet or another source
   - ☐ Miscarriage – **END:** Thank you for participating in the first survey. You do not qualify to participate in this follow-up survey.
   - ☐ Other (please explain): ___________________________ - **END:** Thank you for participating in the first survey. You do not qualify to participate in this follow-up survey.

© 2022 Pleasants EA et al. *JAMA Network Open.*

eTable. Adjusted Odds Ratios for Association Between Selected Distance-Related Barriers to Abortion Access and Distances to Abortion

**Distance-related barrier (OR)**	**<5 miles (n=233)**	**5-24 miles (n=382)**	**p-value**	**25-49 miles (n=85)**	**p-value**	**≥50 miles (n=165)**	**p-value**
Any distance-related barriers reported	(ref)	0.98 (0.60,1.59)	0.925	4.04* (1.20,13.64)	**0.024**	2.07* (1.00,4.27)	**0.049**
The distance I had to travel made if hard	(ref)	1.65* (1.12,2.43)	**0.011**	5.98*** (3.46,10.34)	**<0.001**	8.45*** (5.32,13.41)	**<0.001**
I had to make multiple trips to the facility	(ref)	1.40 (0.98,2.00)	0.061	1.75* (1.04,2.94)	**0.036**	1.98** (1.29,3.03)	**0.002**
I didn't know where to get an abortion	(ref)	1.33 (0.84,2.10)	0.229	2.19* (1.19,4.05)	**0.012**	2.28** (1.37,3.80)	**0.001**
I had to arrange for transport to the facility	(ref)	1.23 (0.86,1.74)	0.253	2.52*** (1.51,4.21)	**<0.001**	2.12*** (1.39,3.23)	**<0.001**
I had to gather money for travel expenses	(ref)	1.07 (0.75,1.51)	0.720	2.64** (1.42,4.93)	**0.002**	1.80* (1.12,2.89)	**0.015**
I had to keep the abortion a secret	(ref)	1.18 (0.85,1.64)	0.324	1.99* (1.18,3.36)	**0.010**	1.62* (1.08,2.44)	**0.021**
I had to arrange for childcare or care for another family member	(ref)	0.86 (0.59,1.25)	0.468	1.71* (1.01,2.89)	**0.044**	1.99** (1.30,3.04)	**0.001**
I had to get time off work/school	(ref)	1.20 (0.86,1.67)	0.276	1.36 (0.83,2.24)	0.224	1.25 (0.84,1.87)	0.272

*p<0.05; **p<0.01, ***p<0.001.

Notes: Models adjusted for state clustering as a random effect.

© 2022 Pleasants EA et al. *JAMA Network Open.*
